# A Model Study on the Role of Wetland Zones in Lake Eutrophication and Restoration

**DOI:** 10.1100/tsw.2001.350

**Published:** 2001-11-27

**Authors:** J.H. Janse, W. Ligtvoet, S. Van Tol, A.H.M. Bresser

**Affiliations:** National Institute of Public Health and Environment (RIVM), Laboratory of Water and Drinking Water Research, Bilthoven, The Netherlands

**Keywords:** shallow lakes, eutrophication, nutrients, nitrogen, phosphorus, wetlands, model, lake restoration

## Abstract

Shallow lakes respond in different ways to changes in nutrient loading (nitrogen, phosphorus). These lakes may be in two different states: turbid, dominated by phytoplankton, and clear, dominated by submerged macrophytes. Both states are self-stabilizing; a shift from turbid to clear occurs at much lower nutrient loading than a shift in the opposite direction. These critical loading levels vary among lakes and are dependent on morphological, biological, and lake management factors. This paper focuses on the role of wetland zones. Several processes are important: transport and settling of suspended solids, denitrification, nutrient uptake by marsh vegetation (increasing nutrient retention), and improvement of habitat conditions for predatory fish. A conceptual model of a lake with surrounding reed marsh was made, including these relations. The lake-part of this model consists of an existing lake model named PCLake[1]. The relative area of lake and marsh can be varied. Model calculations revealed that nutrient concentrations are lowered by the presence of a marsh area, and that the critical loading level for a shift to clear water is increased. This happens only if the mixing rate of the lake and marsh water is adequate. In general, the relative marsh area should be quite large in order to have a substantial effect. Export of nutrients can be enhanced by harvesting of reed vegetation. Optimal predatory fish stock contributes to water quality improvement, but only if combined with favourable loading and physical conditions. Within limits, the presence of a wetland zone around lakes may thus increase the ability of lakes to cope with nutrients and enhance restoration. Validation of the conclusions in real lakes is recommended, a task hampered by the fact that, in the Netherlands, many wetland zones have disappeared in the past.

## INTRODUCTION

As a result of high nutrient loadings during the past decades, many shallow lakes have become highly eutrophic. They are now characterized by dense algal blooms of cyanobacteria, high turbidity, absence of vegetation, and a fish community dominated by bream. Although these effects were caused by high nutrient loadings, restoration of the former macrophyte-dominated clearwater state often could not be achieved by external load reduction alone; eutrophic lakes often show resistance to recovery. Apparently, once the system has switched from a clear to a turbid state, this switch cannot simply be reversed[2,3,4,5]. Several, often interacting, mechanisms for this resistance have been proposed. Firstly, a prolonged internal loading from nutrient-rich sediments may delay the response[6,7]. Secondly, an increase of the nutrient utilization efficiency of the phytoplankton makes them produce the same biomass with less nutrient[8,9]. Thirdly, the grazing pressure on the phytoplankton is low, both because of the poor edibility of cyanobacteria and the strong predation by bream[10]. Finally, the large amount of detritus accumulated in the system, combined with wind/wave action and bioturbation by benthivorous fish, keeps the water turbid and impedes the return of vegetation[11]. Clearly, both direct effects of nutrients and indirect effects through the food web may contribute to the often observed resistance to recovery. Therefore, additional measures are sometimes considered apart from, or combined with, nutrient load reduction[10].

On the other hand, the clearwater state of shallow lakes, dominated by submerged macrophytes, shows a certain resistance to such external forcings as a moderate increase in nutrient loading[12]. Several stabilising mechanisms may play a role. Nutrient uptake by macrophytes may suppress algal growth due to nutrient limitation[13]; they may also provide favourable conditions for predatory fish and reduce wind-induced resuspension by stabilising the sediment.

This paper focuses on the role of wetland zones around lakes and their response to a change in nutrient loading. A number of studies[14,15,16,17] indicate that wetlands, both natural and constructed, may increase the retention of nutrients and other pollutants. Several relations between pelagic and wetland zones may be important in this respect, such as transport and settling of suspended solids, nutrient uptake by the marsh vegetation, and enhanced denitrification, processes all increasing the retention of nutrients. Another type of relation is the role of wetland zones as spawning and nursery areas for predatory fish[18]. A conceptual model of a lake with a surrounding reed marsh was made, which includes these relations in a simplified way (Fig. 1).

The relative area of lake and marsh can be varied in the model, equivalent to the variations in water level occurring in real life.

## METHODS AND PROCEDURES

The study has been performed with a simulation model with two coupled compartments: open water and wetland (Fig. 1). The processes in the coupled model include:

- Transport of particles and nutrients
- Uptake and release of nutrients by the wetland vegetation
- Denitrification
- Relation between wetland zone and predatory fish

PCLake[1] is used as a basis for the open water part, because this model combines a description of the most important biological components (viz. phyto- and zooplankton, fish, and submerged macrophytes) with a description of the nutrient cycles in a shallow lake ecosystem, in both water and top-layer sediment. The model occupies an intermediate space between many eutrophication models, which focus mainly on the nutrient cycle, on the one hand, and more detailed biological models on the other. The model also differs from so-called minimodels[19] in that it is based on closed nutrient cycles, allowing a more quantified analysis.

The structure of the lake model is illustrated in Fig. 2. For a description of PCLake, the model for the lake part, we refer to Janse[1,20]. In short, the model describes the growth of phytoplankton and submerged macrophytes, as well as a simplified food web, coupled to a description of the nitrogen and phosphorus cycles in the water column and the sediment top layer. The nutrient cycles are closed within the model system.

The wetland part of the coupled simulation model is composed of a simplified growth model for reed[21,22,23] coupled

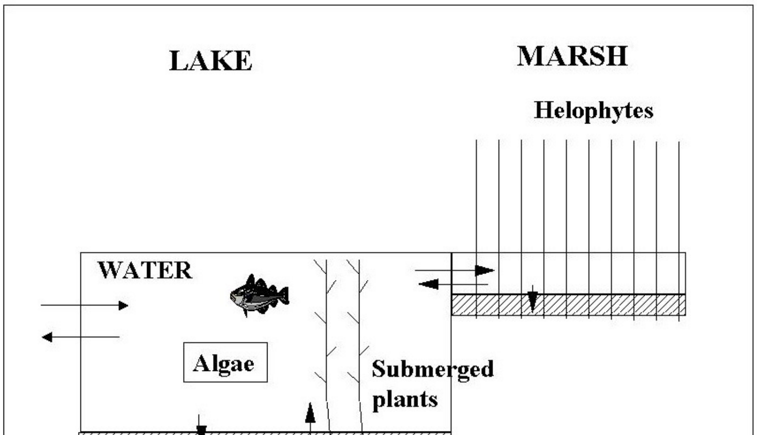

**FIGURE 1**. Lake-wetland system.

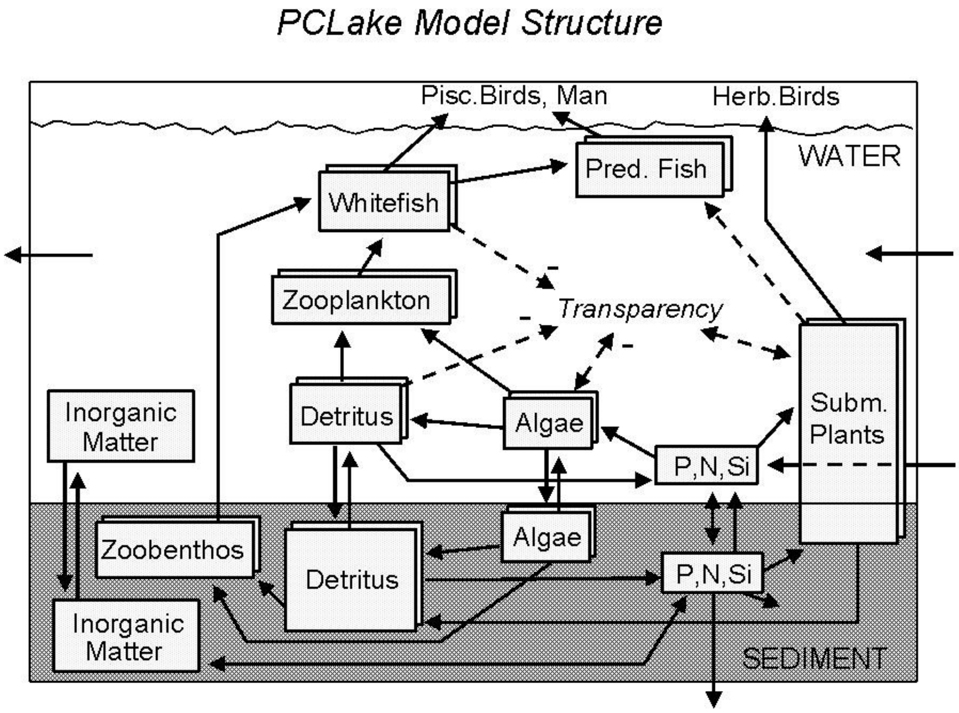

**FIGURE 2**. Model structure of PCLake.

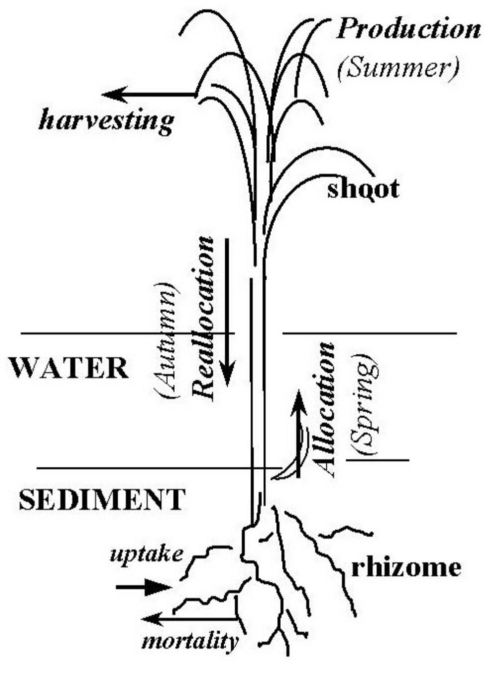

**FIGURE 3.** Model of wetland vegetation.

to a description of the nutrient processes in the water column and the sediment top layer of the marsh zone[15, 24] (Fig. 3). A description with references of the processes and the parameter values used is given by Van Tol[25] and will be published elsewhere. The main features are described here.

The biomass of the marsh vegetation is divided into root and shoot fractions. The seasonal development is modelled as allocation of a part of the root biomass to the shoots in spring, photosynthetic growth during summer, and partial reallocation back to the roots in autumn. Summer growth is assumed to be dependent on the marsh water depth, N and P in the sediment top layer, daylight, and temperature. Nutrients are taken up from the sediment top layer only. Optionally, regular mowing of the vegetation can be taken into account (in the current study, mowing is not included).

The substances and process descriptions are analogous to those in the lake model, except that the water depth is much lower (default 0.5 m), settling velocities are higher due to the absence of wind action, and resuspension is assumed to be zero. Phytoplankton is assumed not to grow in the shadow of the reed vegetation.

Mixing between the water columns of the lake and the wetland is described by a dispersion-like equation across the contact zone:

$$F = k_{exch} * A/L * (c_{lake} - c_{marsh})$$
$$\approx k_{exch} * D_{marsh}/(0.5 * fMarsh) * (c_{lake} - c_{marsh})$$

in which F is the mass flux between lake and marsh (g day$^{-1}$), $k_{exch}$ the exchange coefficient, comparable to a dispersion coefficient (m$^2$ day$^{-1}$), A the interfacial area (m$^2$), L the mixing length (m), $D_{marsh}$ the marsh water depth (m), fMarsh the relative marsh area (–), and $c_{lake}$ and $c_{marsh}$ the concentrations (g m$^{-3}$) of a substance in the lake and the marsh water, respectively. The exchange coefficient is by default set to 10$^5$ m$^2$ day$^{-1}$, corresponding to efficient mixing. Lower coefficients have also been used in the simulations, however (see below).

The relation between the wetland zone and fish population is simplified to the role as spawning and nursery area for predatory fish. It is assumed that the maximum possible biomass of these fish increases, within certain limits, with the relative area of wetland vegetation[18]:

$$B_{max} = MIN\ [B_{max,\ min} + B_{rel} * 100 * fMarsh,\ B_{max,\ max}]$$

in which $B_{max}$ is the maximum stock (carrying capacity) of predatory fish, $B_{max,\ min}$ and $B_{max,\ max}$ the minimum (4 kg fish ha$^{-1}$) and maximum (75 kg ha$^{-1}$) carrying capacity, respectively, and $B_{rel}$ the relative increase of $B_{max}$ per % marsh. This last parameter amounts to about 5 kg ha$^{-1}$ %$^{-1}$, or 7 in case submerged plants are also abundant in the lake.

The model has been implemented in the simulation package ACSL/Math v. 2.4 (which includes ACSL v. 11). So far, the lake model has already been used for several scenario analyses. These include studies on nutrient load reduction[20], biomanipulation[26], and combinations of these with dredging[27,28,29]. A partial calibration study on a multilake data set using Bayesian statistics has also been carried out[30]. The coupled lake-wetland model has not yet been calibrated on observed data.

With this coupled model, the question is studied: how is the water quality in shallow lakes (expressed as nutrient concentrations, Secchi depth, and chlorophyll-*a*) influenced by the presence and relative size of a wetland area along the lake?

The simulations have been carried out with starting conditions typical for Western European shallow lakes, i.e., turbid water, sediments heavily loaded with nutrients, and a fish stock dominated by benthivorous and zooplanktivorous fish. The most important assumptions in the simulations are:

- Depth of the lake: 2 m.
- Lake surface area: 200 ha.
- Water depth in the wetland zone: 0.5 m.
- A constant (i.e., not variable in time) water and nutrient influx to the lake. The water influx was set to 10 mm day$^{-1}$, i.e., a retention time of just over 0.5 year.
- Influx to the marsh zone takes place via the lake only.
- No mowing of the wetland vegetation.

The following input factors were varied in the simulations:

- The size of the wetland area relative to that of the lake (called fMarsh) was varied between 0 and 1 (–).
- The amount of nutrient loading was also varied. The nominal total N loading was set at 0.024 g m$^{-2}$ day$^{-1}$ and the P loading at 0.0022 g m$^{-2}$ day$^{-1}$. For the sensitivity analysis, the N loading was varied between 0.009 and 0.029 g m$^{-2}$ day$^{-1}$, with the P loading 20% of these values (making nitrogen the limiting nutrient).
- Three levels of mixing rate between the open water and the water in the wetland part were used: besides intensive mixing (exchange coefficient = 10$^5$ m$^2$ day$^{-1}$), the nominal setting, lower values of 10$^4$ and 10$^3$ (m$^2$ day$^{-1}$) were used.

The simulations have been carried out for a period of 10 years with a variable time step of integration. Weather conditions in this period (temperature, daylight) are assumed to follow sine curves, with the average ranges taken from the period 1961–1990. Additional simulations were done excluding the wetland-fish relation in order to compare the contributions of the different processes.

## RESULTS AND DISCUSSION

Simulations of the development over time over time of some important variables, for different sizes of the wetland area and with nominal values for loading and mixing, are shown in Figs 4, 5, 6, 7, 8, and 9. For each figure, N loading = 0.024 g m$^{-2}$ day$^{-1}$, P loading = 0.0022 g m$^{-2}$ day$^{-1}$, and $k_{exch}$ = 10$^5$ m$^2$ day$^{-1}$.

The aboveground marsh vegetation (g d.w. m$^{-2}$; Fig. 4) gradually increases over the years. The biomass per m$^2$ reached is somewhat lower when the wetland area increaes, due to nutrient dilution effects; the total biomass marsh vegetation in the area (biomass per m$^2$ times the area) is, of course, much larger. For nutrient concentrations (total N: Fig. 5; total P: Fig. 6), the model shows a substantial decrease of both nutrient concentrations at increasing wetland area.

As you can see in Fig. 7, a substantial decrease of summer peaks in chlorophyll-*a* occurs only at wetland areas of 0.5 times the lake area or more. A substantial increase in submerged vegetation (Fig. 8) only occurs at areas of 0.5 times the lake area or more. At a relative area of 1.0, a stable macrophyte development is simulated, while at a value of 0.5 the development is still unstable.

In agreement with the model assumptions, a reasonable wetland size (>10% of the lake) as breeding habitat is essential for the development of a substantial stock of predatory fish (Fig. 9).

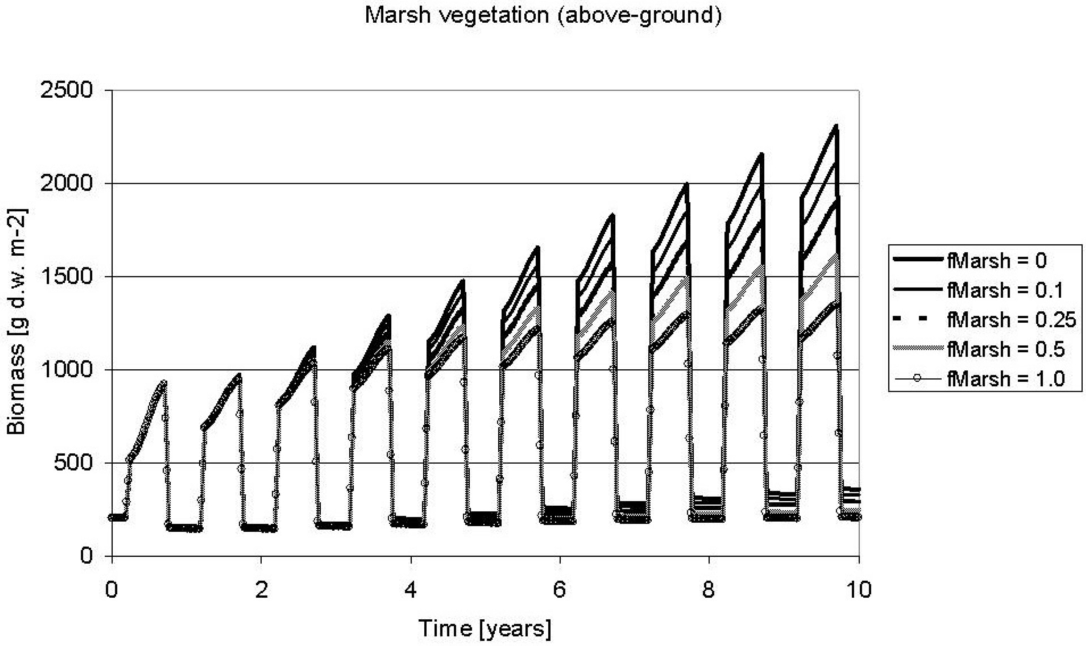

**FIGURE 4.** Marsh vegetation.

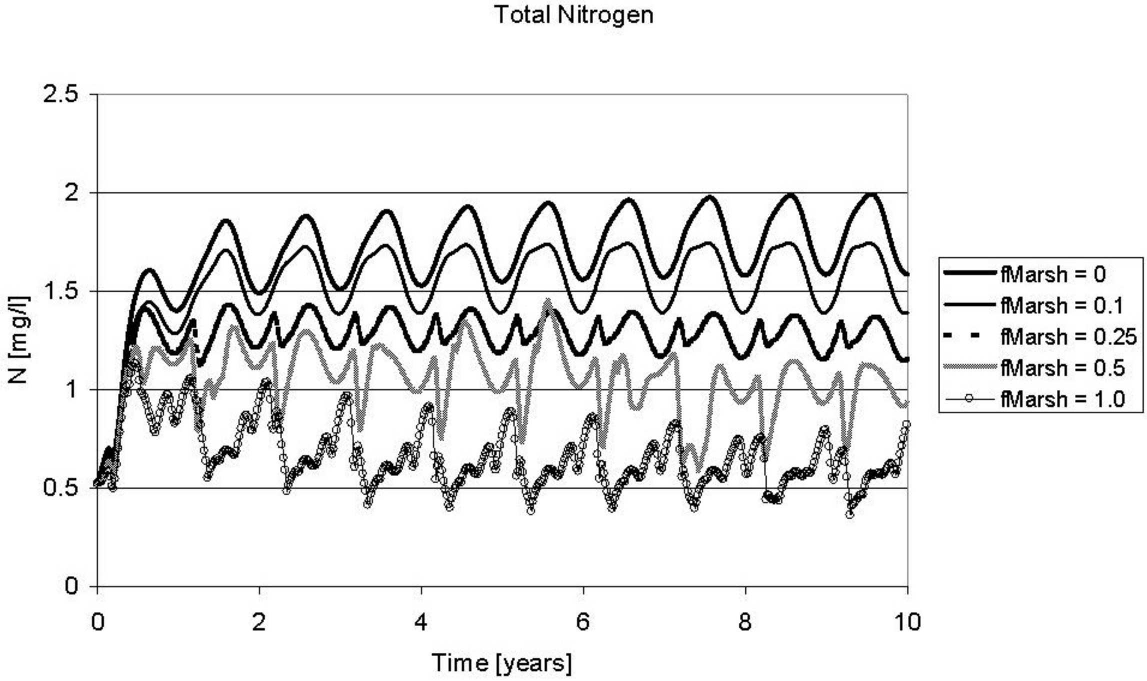

**FIGURE 5.** Total N.

At large marsh areas, the stock again decreases because of nutrient dilution effects.

The relative importance of the predatory fish relations (i.e., the wetland as breeding habitat) in the model was demonstrated by additional simulation runs with this relation omitted from the model. For a relative wetland area of 0.5 times the lake area, for instance, the slowly starting macrophyte development as shown in Fig. 8 vanishes and the chlorophyll-*a* concentration remains high if this relation is omitted (graph not shown), showing that the fish relation indeed contributes to the effect.

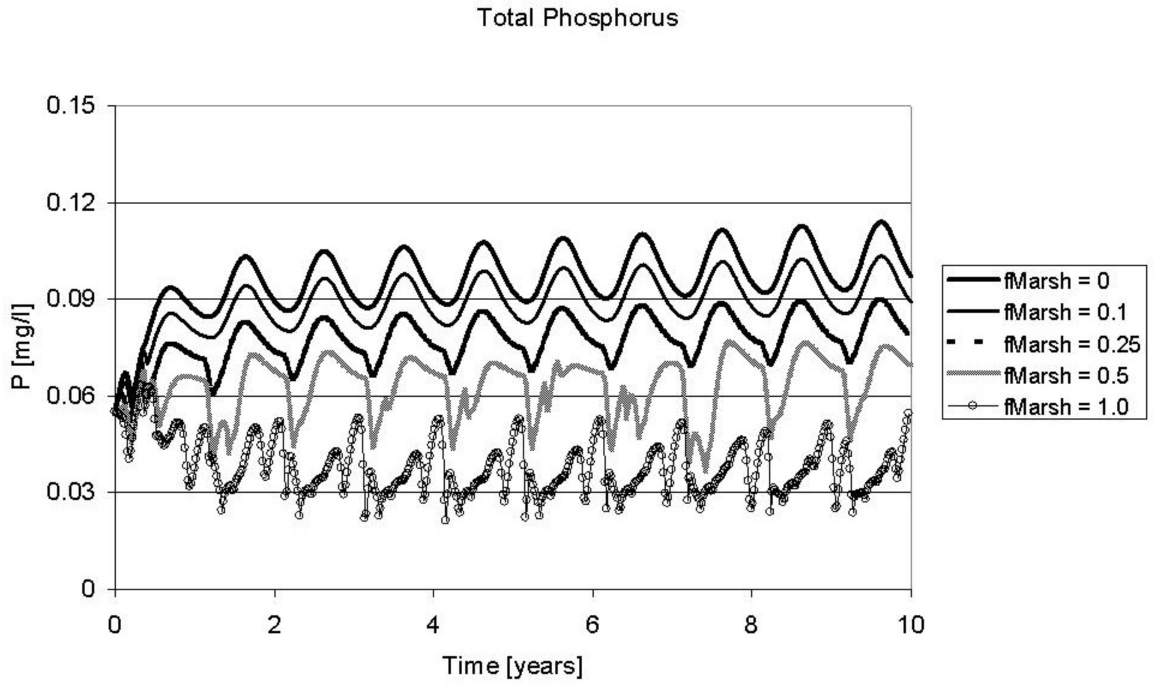

**FIGURE 6.** Total P.

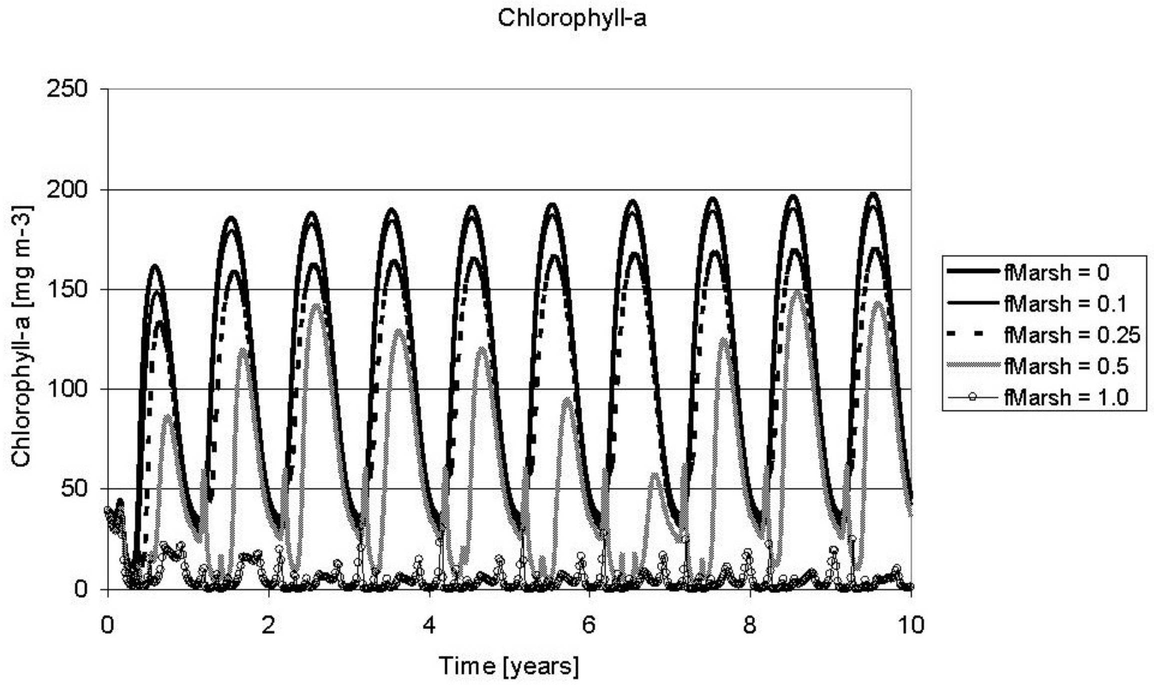

**FIGURE 7.** Algal biomass.

The effects of increasing marsh area shown in Fig. 4–9 are observed only if the nutrient loading is not too high (Fig. 10a–c). The critical nitrogen loading level increases with increasing wetland area. Comparable results are obtained for the phosphorus loading if this nutrient, rather than nitrogen, is made limiting. Furthermore, the effects are clearly dependent on the mixing rate between lake and marsh water. While at the high value of the exchange coefficient of $10^5$ $m^2$ $day^{-1}$ clear effects of the marsh are observed (Fig. 10a), they become less apparent or even disappear in case of intermediate ($10^4$; Fig. 10b) or low ($10^3$; Fig. 10c) mixing rates. At the high value of $10^5$, there

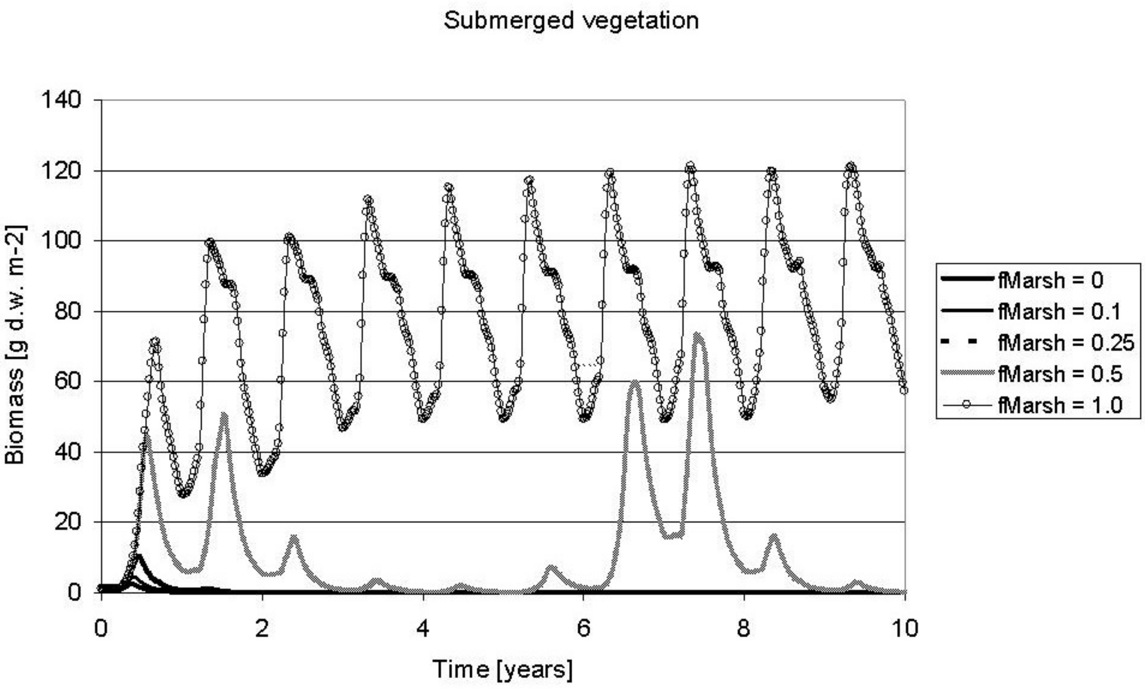

**FIGURE 8.** Submerged vegetation.

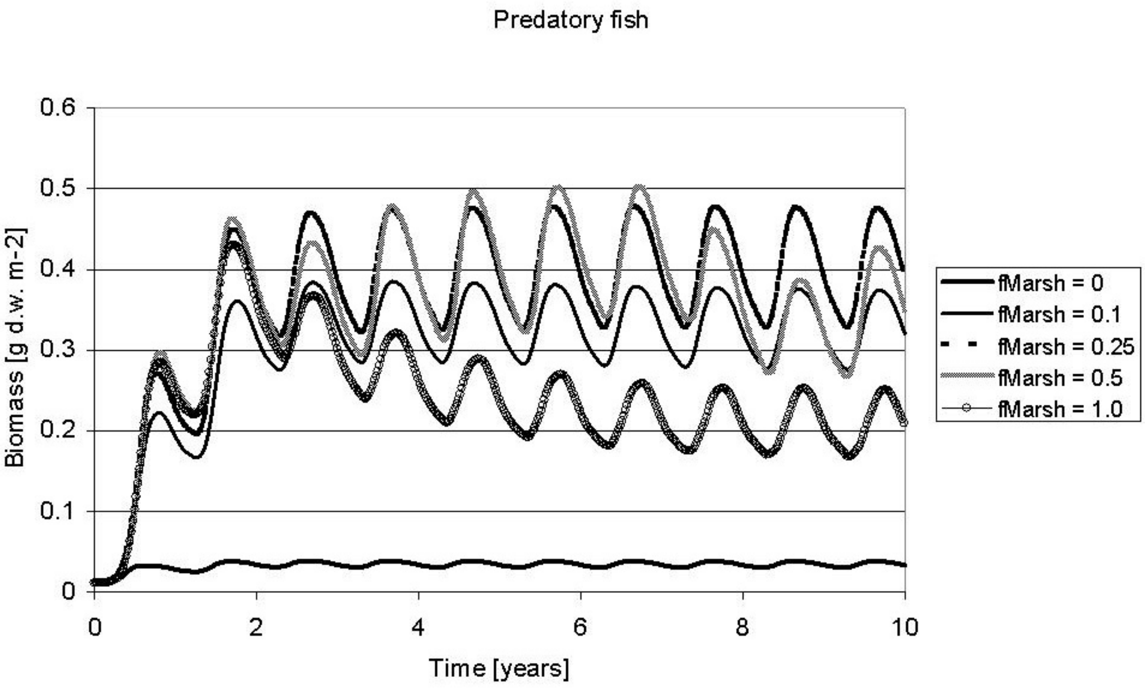

**FIGURE 9.** Predatory fish biomass.

is little difference in the simulated concentrations of substances between the water columns in the lake and the marsh.

Comparing the simulated year-average N and P concentrations with the nutrient input, for the nominal run, the retention of nitrogen in the system increases from 25% in the lake without marsh, to 34, 47, 58, and up to 76% for the largest marsh area. For phosphorus, the retention started higher (52%) in the lake proper, and increased somewhat less with the marsh area: 57, 62, 69, and up to 83%. This is for an N/P ratio in the input of >10 (g/g); for other ratios and loading levels, the percentages differ, but the trend remains. The extra retention of nitrogen by the marshland relative to phosphorus is probably due to the extra contribu-

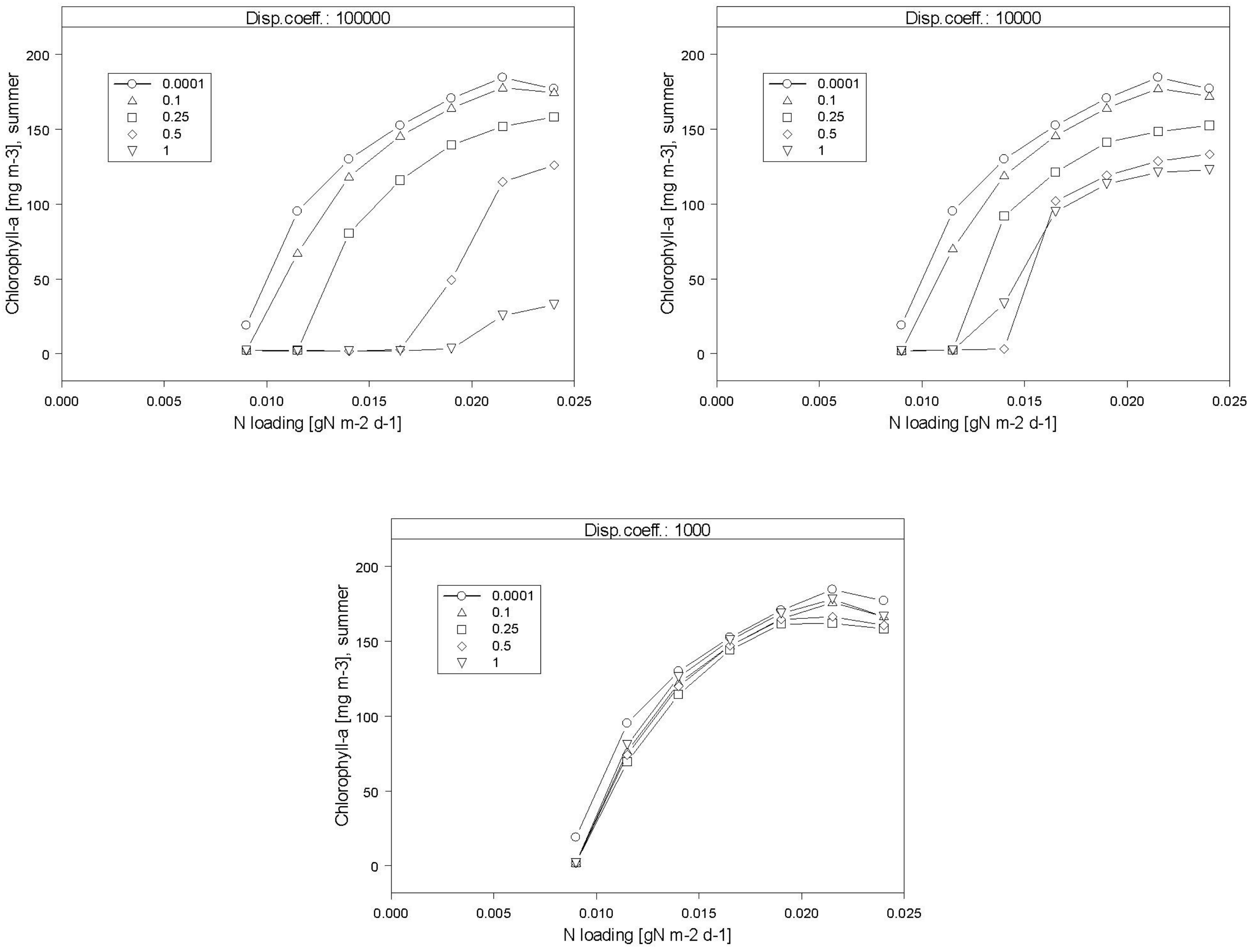

**FIGURE 10.** Simulated average chlorophyll-*a* concentrations as a function of nitrogen loading and marsh area, for three values of the exchange coefficient.

tion of denitrification in the wetland area. The annual amounts of nutrients retained are in the range of 1 to 10 g N $m^{-2}$ $year^{-1}$ and 0.1 to 1 g P $m^{-2}$ $year^{-1}$, depending on the input settings. The study indicates that the modelled wetland system could play a significant role in lake restoration only if the area is relatively large, the water exchange is good, and the nutrient loading to the lake is not too high.

The retention values are in the range of those reported[15] or modelled[31] in many studies for natural wetlands, although lower and higher values are also found[15,32]. The values for different systems are often difficult to compare because of large differences in loading, physical characteristics, and hydrological regime. Values are generally higher for constructed wetlands[14,15,33] where dimensions are set to be optimal for water purification.

Translation of the simulated effects to those in real-world lake systems cannot be done automatically. In specific situations, one should verify the assumptions made in relation to the actual circumstances. For instance, the systems' response may differ in case of fluctuating water tables, or if the marsh is an instream wetland. Finally, one should of course find a balance between the use of natural wetlands for lake purification, and the risk of possible damage to the actual ecological values of the wetlands[34].

## CONCLUSIONS

The simulations clearly show the extra purification effect of a lake with a substantial wetland zone as compared to lakes without such a zone. There is a positive relation between nutrient removal and the size of the wetland area. The switch from phytoplankton-dominated systems to lakes with submerged vegetation is easier with wetlands present, but only if the size of the marshes is substantial, the nutrient loading is moderate, and sufficient mixing is present. Transport and sedimentation processes seem to be the most important processes, but the food-web relations via predatory fish also play an important role, especially at intermediate nutrient conditions. The model takes a stabilisation time of several years, especially for the development of emergent vegetation.

The overall performance of the model is promising. Calibration of the model has been possible up to now mainly with data from rather eutrophied situations. Expansion of the available data set with time series from mesotrophic lakes is essential. Validation with an independent data set is foreseen.

Besides the mere wetland size, the effects of variations in hydrologic regime and water tables both in the lake and in the wetland area should also be studied. A more extensive sensitivity analysis on model parameters and lake characteristics also has to be carried out. With these additional analyses on the behaviour of lake-wetland systems, the model may play a role in the development of more integrated lake and catchment management strategies.

| | | |
|---|---|---|
| Received: | July | 21, 2001 |
| Revised: | October | 31, 2001 |
| Accepted: | October | 1, 2001 |
| Published: | November | 27, 2001 |
